# Genetic variation for resistance to the specific fly pathogen *Entomophthora muscae*

**DOI:** 10.1038/s41598-020-71262-w

**Published:** 2020-08-31

**Authors:** Jonathan B. Wang, Carolyn Elya, Raymond J. St. Leger

**Affiliations:** 1 Department of Entomology, University of Maryland, College Park, MD 20742 USA; 2 Department of Organismic and Evolutionary Biology, Harvard University, Cambridge, MA 02138 USA

**Keywords:** Biological techniques, Pathogenesis

## Abstract

We found substantial variation in resistance to the fly-specific pathogen *Entomophthora muscae* 'Berkeley' (Entomophthoromycota), in 20 lines from the *Drosophila melanogaster* Genetic Reference Panel (DGRP). Resistance to *E. muscae* is positively (r = 0.55) correlated with resistance to the broad host range ascomycete entomopathogen *Metarhizium anisopliae* (Ma549), indicative of generalist (non-specific) defenses. Most of the lines showing above average resistance to Ma549 showed cross-resistance to *E. muscae*. However, lines that succumbed quickly to Ma549 exhibited the full range of resistance to *E. muscae*. This suggests fly populations differ in *E. muscae*-specific resistance mechanisms as well as generic defences effective against both Ma549 and *E. muscae*. We looked for trade-offs that could account for inter-line variation, but increases (decreases) in disease resistance to *E. muscae* are not consistently associated with increases (decreases) of resistance to oxidative stress, starvation stress and sleep indices. That these pathogens are dynamic agents of selection on hosts is reflected in this genetic variation for resistance in lines derived from wild populations.

## Introduction

Considerable genetic variation in resistance and tolerance to infection can exist within populations^1,2^. This variation determines the burden of disease, and represents the raw material from which populations can evolve resistance either naturally or artificially (i.e. through selective breeding by humans)^3^. Insects are no exception to this pattern, and the same population of *Drosophila melanogaster* can contain resistant and susceptible genotypes to viruses, fungi and bacteria^4,5^.

Many arthropod pathogenic fungi belong to the phylum Entomophthoromycotina and most of the remainder are distantly related ascomycetes^6^. We previously demonstrated significant variation in the life-span of 188 *Drosophila* Genetic Reference Panel (DGRP) lines infected with the ascomycete fungus *Metarhizium anisopliae* ARSEF 549 (Ma549)^5^. In addition, we found that resistance to Ma549 was correlated with resistance to the bacterium *Pseudomonas aeruginosa* (Pa14), and several previously published DGRP phenotypes including oxidative stress sensitivity, starvation stress resistance, hemolymph glucose levels, and sleep indices. As bacteria infect per os and fungi through the cuticle, the cross-resistance to Pa14 and Ma549 is suggestive of general (multipurpose) humoral defense mechanisms that do not involve cuticle or gut immunocompetence. Consistent with this, a genome-wide association study revealed a network of Pa14 and Ma549-resistance genes that are functionally connected through many different aspects of host physiology^5^. These observations are in line with insertional mutagenesis results: Lu^7^ reported that 87% of mutated genes in more susceptible *Drosophila* lines are involved in a broad spectrum of biological functions not connected with canonical immune systems. The large numbers of pleiotropic genes involved in resistance to Ma549 and Pa14 contrasts with the small number of common polymorphisms associated with resistance to viruses^4^. Interestingly, each viral resistance SNP was associated with resistance to only one virus, which suggests that viral immunity is mediated by a suite of specific factors^4^.

Most models of hosts and pathogens assume there is a tight relationship of co-evolved interactions between species pairs^8^. Such hosts and parasites are thought to engage in antagonistic coevolution, where a newly evolved parasite virulence mechanism is negated over time by a newly evolved host immune mechanism and vice versa^9^. The broad host range of *Metarhizium* and *Beauveria* spp. used in several previous studies on *Drosophila*^10–13^ suggest that these pathogens have not engaged in a strict coevolutionary arms race with *Drosophila*^14^. As study systems, these microbes will not, therefore, tell us about how specialist parasites suppress host immunity, or about any secondary immune mechanisms hosts deploy against specialist parasites^15,16^.

Given the importance of the *Drosophila* model system to our understanding of immunity, it is surprising that very little is known about its natural parasites. There may be 5.5 million insect species^17^, and if every metazoan species has at least one host-specific parasite as some studies suggest^18^, narrow host range entomopathogenic fungi may exist by the millions as well. However, an ecologically relevant specialist fungal pathogen of *Drosophila* pathogen that would facilitate understanding of host pathogen evolution and identify specialized immune mechanisms has only recently been identified^19^. Behavior-manipulating fungal pathogens in the *Entomophthora muscae* (Entomophthoromycota) species complex are best known for causing epizootic outbreaks in house flies. However, Elya et al.^19^ identified an epizootic in Californian *Drosophila* caused by a single strain of *E. muscae* (*E. muscae* 'Berkeley'). It remains unclear if *E. muscae* 'Berkeley' is a distinct lineage (or even species) from those that infect other fly species, and how specific it may be for *Drosophila* spp. over other dipterans is also unknown^19^. However, contrary to other fungal infections (e.g., *Metarhizium*), and consistent with previous *E. muscae* infections described in house flies^20^, *E. muscae* 'Berkeley' invaded the *Drosophila*’*s* nervous system and caused a characteristic set of behaviors: on their final day of life, a few hours before sunset, the flies climb upward, extend their proboscides, affixing them to a substrate, then raise their wings, clearing a path for infectious spores to launch from their abdomens^19^. This robust control of behavior by *E. muscae* 'Berkeley' indicates a high level of adaptation of the pathogen to the host. However, many aspects of this disease (e.g., the climbing behavior of critically ill hosts), are typical for narrow host range pathogens of arthropods, and probably involve the pathogens taking advantage of sleep behavior in insects, as these behaviors are highly conserved^21^. Many of the best characterized and most commonly witnessed epizootics are caused by behavior-modifying entomophthoralean species infecting flies, ants, grasshoppers, caterpillars and cicadas^22,23^.

In this study we bioassayed *E. muscae* 'Berkeley' (hereafter referred to as *E. muscae*) against a subset of 20 DGRP lines selected because they represent the genotypes that are the most resistant or susceptible to Ma549 from the DGRP collection. Using this divergent subset, we show that wild-derived populations of *Drosophila* have substantial differences in susceptibility to *E. muscae*, and that this variation correlates with resistance to Ma549, and, to a lesser extent, with starvation resistance and sleep indices. However, lines that succumbed quickly to Ma549 covered the whole spectrum of resistance to *E. muscae* from low to high. This suggests there are additional mechanisms by which disease resistance to *E. muscae* can be altered, besides those effective against Ma549.

## Methods

### Divergent DGRP lines

DGRP Freeze 2 lines, originally derived from an out-crossed population in Raleigh, North Carolina, by the Mackay laboratory^24^ were obtained from the Bloomington Stock center. To characterize natural variation in susceptibility to *E. muscae,* we used a subset of the 188 DGRP lines deployed in Wang et al.^5^, comprising the 10 most Ma549 resistant and 10 most Ma549 susceptible DGRP lines. Called the “divergent subset” in Wang et al.^5^, they represent the most extreme disease phenotypes to Ma549 in the DGRP. Ma549 and Pa14 LT_50_ data for the divergent subset was previously published in Wang et al.^5^.

### *E. muscae* exposure of divergent DGRP lines

All flies were reared on cornmeal-based diet (3% weight per volume (w/v) cornmeal, 11% w/v dextrose, 2.3% w/v yeast, 0.64% w/v agar and 0.125% w/v tegosept) at 21 °C on a 12:12 light:dark cycle. For infection, we followed a modified version of the protocol described in Elya et al.^19^ using *E. muscae* that has been propagated in *Drosophila *in vivo since 2015. Briefly, 21 “exposure vials” were prepared, each by embedding six Canton-S *Drosophila* cadavers freshly killed by *E. muscae* headfirst into minimal media containing 5% sucrose and 1.5% agar in wide fly vials (Genesee Scientific). For each of the 20 DGRP lines and Canton-S, fifty flies (25 male and 25 female) aged < 5 days post eclosion were transferred to a fresh vial and the plug of the vial was pushed down to confine the flies within 2 cm to improve the likelihood that they would encounter infectious spores. Vials were housed for the first 24 h under high humidity at 21 °C with a 12:12 light:dark cycle, at which point the plug was raised to relieve fly confinement. Flies were housed at 21 °C with ~ 40% humidity for the remainder of the experiment. Each vial was monitored twice daily (once before subjective sunset, once after) for deaths and subsequent sporulation, to confirm death by *E. muscae*. All experiments were replicated five times, the raw data is provided in Supplemental Table S1.

### Data analysis

All statistics were done using R version 3.6.1. To determine the relationship between different phenotypes, we performed Pearson correlations using the package Hmisc. We tested for normality using Shapiro-Wilks test. Comparisons between sexes and *Wolbachia* infection statuses were done using the non-parametric two-sided Mann–Whitney test.

## Results and discussion

To characterize natural variation in pathogen resistance, we quantified susceptibility to *E. muscae* using the divergent subset of the 10 most and the 10 least Ma549 resistant DGRP lines (selected out of 188 DGRP lines). Age-matched flies from each line were exposed to *E. muscae*, and survival time was monitored using five replicates (25 flies each), per sex per line. Elya et al.^19^ report that the Wolbachia-free CantonS *Drosophila* developed a strong immune response one day after infection with *E. muscae* but by the third day the fungus had spread throughout the body, and most flies died around four to five days following infection. Unlike Ma549 and Pa14, *E. muscae* consistently kills hosts at the same zeitgeber time every day (always in the hours leading to subjective sunset), therefore, we used the percentage surviving at five days post-exposure to *E. muscae* (referred to as a PS_5_; daily deaths only rarely peak after this time point) as our metric to compare to Ma549 and Pa14. In contrast to *E. muscae*, DGRP lines die from Ma549 or Pa14 at different rates over a broader range of day’s post-exposure, so post-infection survival for these pathogens is better measured using LT_50_ values.

**Figure 1:**
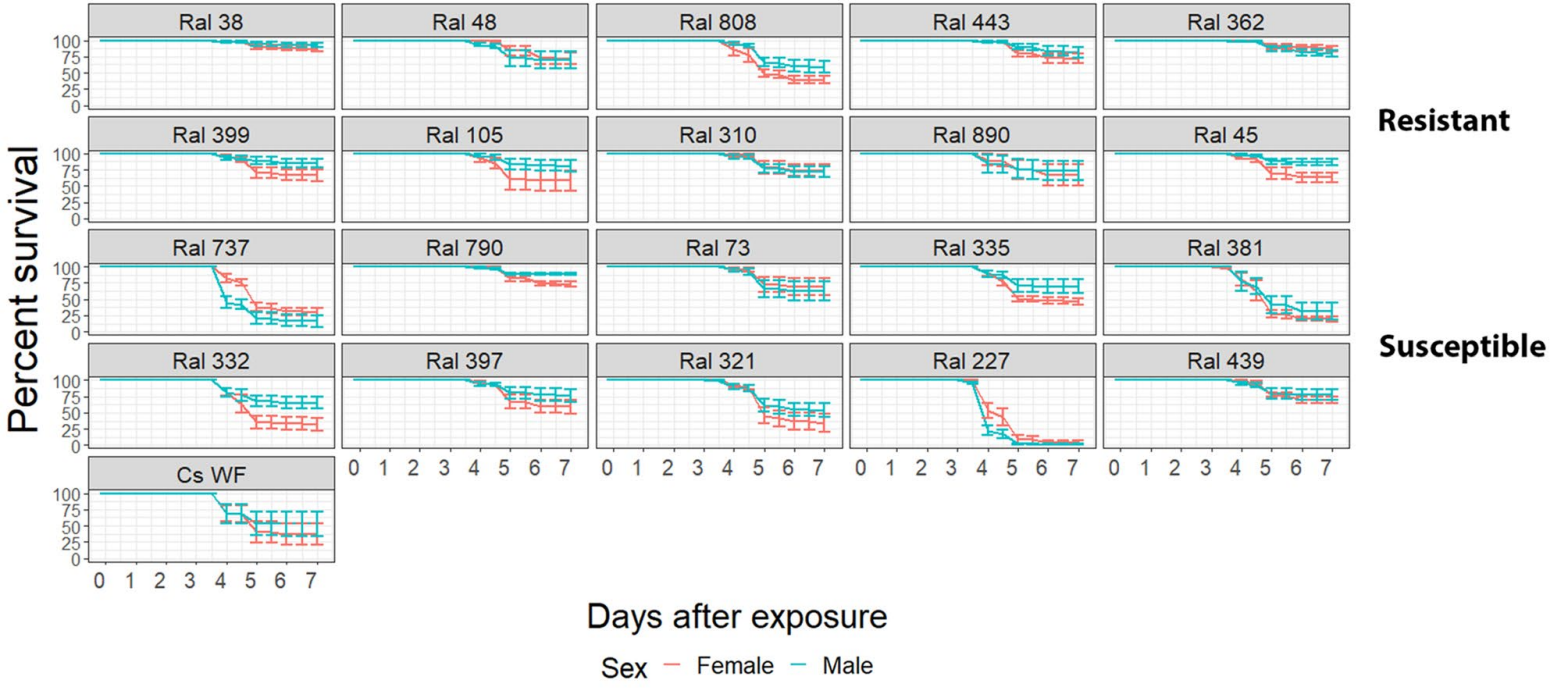
Percent survival of DGRP lines tested with *E. muscae.* Flies of the divergent subset were broken up into two groups, those resistant to *Metarhizium anisopliae* (top two rows) and those susceptible to *M. anisopliae* (middle two rows). Canton-S flies (CS WF) (bottom) used previously to establish that *E. muscae* 'Berkeley' is a *Drosophila* pathogen^19^ were used as a positive control. Percentages are an average of five replicates and error bars reflect standard errors.

Using the DGRP lines, we show that wild-derived populations of *Drosophila* have substantial differences in susceptibility with mortalities ranging from 1.6 to 94%, and a mean survival of males (females) of 70% (62%) (Fig. 1). Less than 25% of flies in the most *E. muscae*-resistant DGRP lines had died seven days post exposure, and most of those that succumbed did so after 4 to 5 days. At the other extreme, almost 100% of RAL 227 flies were dead at five days post exposure. After five days the death rate plateaued off for most lines, and approached that of uninfected flies, suggesting that the survivors had cleared the infection. Thus, variable host susceptibility is illustrated by some DGRP lines dying more than others four to five days post infection with *E. muscae*.

**Figure 2:**
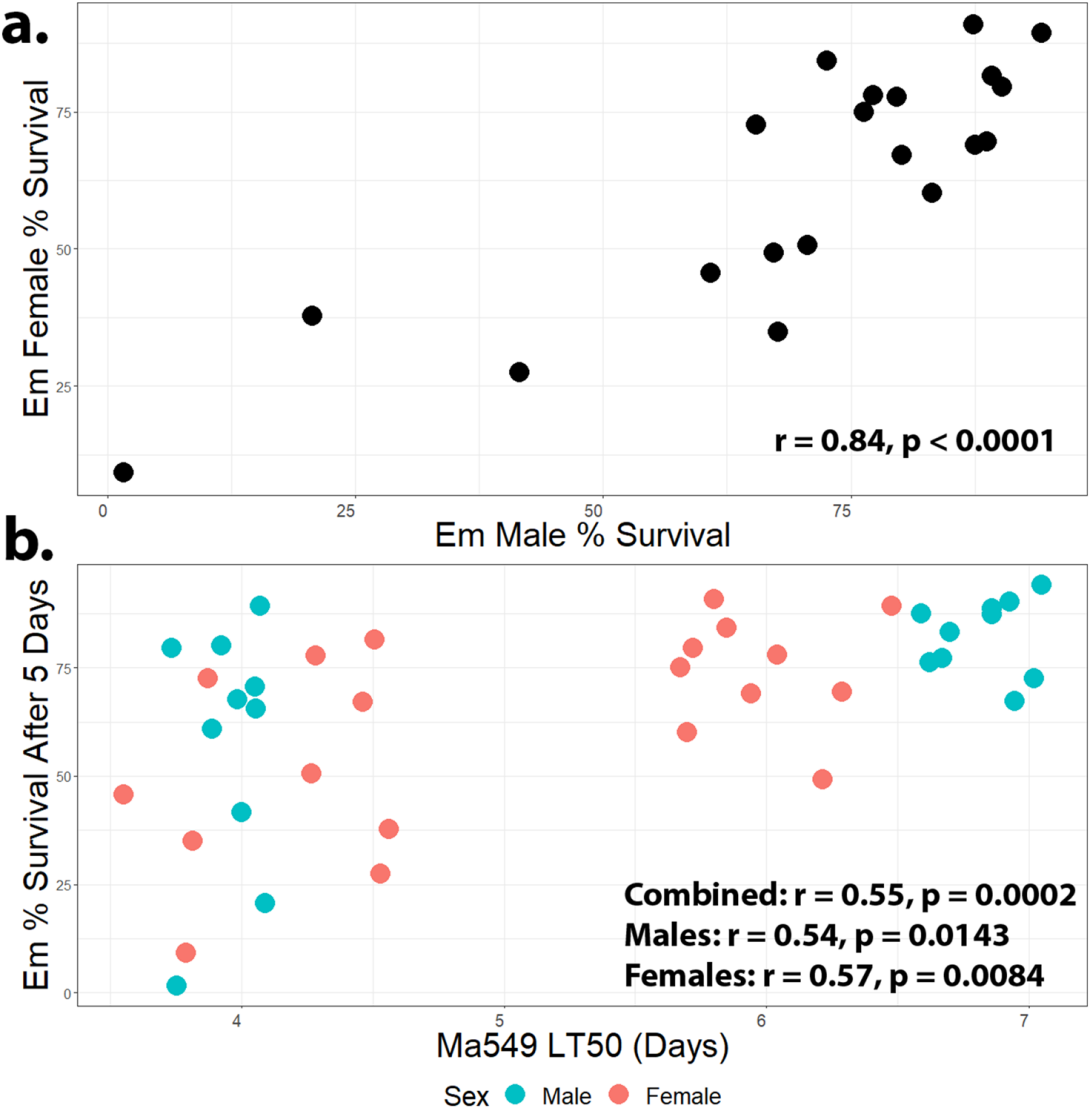
Correlation graphs. Positive correlations of (**a**) % survival of male and female DGRP flies 5 days post-infection with *E. muscae*, and (**b**) between flies infected with *E. muscae* (% survival) or *M. anisopliae* (Ma549 LT_50_ values). Ma549 LT_50_ values were obtained from Wang et al. 2017^5^.

To identify general (multipurpose) defense, the PS_5_ for males and females exposed to *E. muscae* was plotted against average LT_50_ for males (females) infected with Ma549 (Fig. 2b). The data on Ma549 and Pa14 is derived from our earlier publication which used replicates run on different days to randomize environmental variation^5^. Correlations were moderate but highly significant (r = 0.54, 0.57, *p* = 0.0143, 0.0084 for males, females respectively), consistent with *Drosophila* utilizing unspecific generalized defense components against *E. muscae* and Ma549 (Fig. 2b). We previously reported that LT_50_ values for Ma549 and *P. aeruginosa* Pa14 were correlated for both males (r = 0.45, n = 78) and females (r = 0.40, n = 78)^5^. Correlations between Ma549 and Pa14 in the divergent subset used to assay *E. muscae* were greater, with r = 0.7 for males (*p* = 0.0024, n = 16) and r = 0.55 for females (*p* = 0.0262, n = 16) (Fig S1b). Although correlations were still positive between *E. muscae* and *P. aeruginosa*, they were not significant for males (r = 0.09, *p* = 0.7295, n = 16) or for females (r = 0.39, *p* = 0.138, n = 16) (Fig S1a). Ma549 is a broad host range generalist insect pathogen, while Pa14 is a human clinical isolate, and so represents a novel association that will have no history of coevolution. Our results suggest that the genetic basis for resistance to a non-coevolved bacterium (Pa14) and an opportunistic broad host range fungus, share more genetic causes than Pa14 and *E. muscae*.

The weak correlations indicate that *Drosophila* has alleles with pathogen-specific effects to *E. muscae*. This is consistent with variation in the magnitude and direction of association between Ma549 and *E. muscae*. We found greater variation in susceptibility to *E. muscae* among the lines susceptible to Ma549 (male range: 1.6–76.28%, female range: 9.18–84.23%) compared to lines resistant to Ma549 (male range: 77.25–94.2%, female range: 60.13–90.83%) (Fig. 1). Except for RAL 808, the lines resistant to Ma549 were also resistant to *E. muscae*, while lines that succumbed quickly to Ma549 covered the spectrum of resistance to *E. muscae* from low to high (Fig. 1). The exception, RAL 808, is the third most resistant line to Ma549, but is moderately susceptible to *E. muscae* ranking fifteenth out of the twenty lines (PS_5_ ~ 50%). These results suggest that there are multiple mechanisms by which disease resistance to *E. muscae* can be altered besides those effective against Ma549. The shared history of *E. muscae* and *D. melanogaster* could have resulted in a co-evolutionary process that altered the diversity of resistance genes compared to naïve pairs of hosts and pathogens. Similarly, host–pathogen coevolution increases genetic variation in susceptibility to viruses^25^. Thus, heritable variation for host resistance was detectable for two natural viruses of *D. melanogaster*, but not for two non-natural viruses^4^.

To identify sexual dimorphism, we measured disease resistance separately for males and females infected with *E. muscae* (Fig. 2a). Cross-sex genetic correlations were high (r = 0.84, *p* < 0.0001, n = 20), indicating that many of the same variants affect *E. muscae* resistance in males and females. Females flies died more quickly than males when infected with Ma549^5^ (*p* = 0.00039, n = 188). This difference is not significant overall for females of the divergent set (*p* = 0.37, n = 20), but females group separately from males in the most resistant DGRP lines (Fig. 2b). Females were also slightly more susceptible than males to *E. muscae* though this fell short of significance (*p* = 0.2, n = 20). As observed previously for Ma549^5^, RAL 737 was exceptional, as females of this fly line were more resistant to *E. muscae* than males (Fig S2).

*Wolbachia pipientis* is a natural intracellular symbiont of many arthropods, and *Wolbachia* may confer protection against the fungus *Beauveria bassiana* in one *D*. *melanogaster* line^26^. *Wolbachia* status in the DGRP lines was without significant effect on susceptibility to Ma549^5^. Eleven of the twenty divergent lines were positive for Wolbachia, seven of these eleven were present in the ten most susceptible lines producing no significant effect on the susceptibility to *E. muscae* for males (*p* = 0.15, n = 20 or females *p* = 0.71, n = 20).

Resistance to multiple pathogens should have a selective advantage unless this general defense is traded off against other (pathogen-independent) fitness components^27^. In the absence of such a trade-off, directional selection would presumably lead to fixation of genotypes showing general resistance. Table S2 shows the divergent subset, and their life cycle parameters and rankings in publicly available data from other publications, including our data for Ma549 and Pa14. Figure S1 presents correlations between the disease resistance phenotypes in our studies and these other traits. The small sample size (n = 20) of the divergent set reduces the discriminatory power of correlation analysis. However, r values for the divergent set and the total population (188 lines) are similar for many phenotypes. For example, correlations between female resistance to Ma549 and paraquat (a source of oxidative stress) are r = 0.46, *p* = 0.0541 (divergent set, n = 18) and r = 0.31, p < 0.0001, n = 156 (total population), and correlations between female resistance to Ma549 and negative geotaxis are r = 0.29, *p* = 0.2411 (divergent set, n = 18) and r = 0.2, p < 0.0079, n = 171 (total population). The corresponding values for *E. muscae* are r = 0.25, *p* = 0.325, n = 18 (paraquat resistance) and r -0.04, *p* = 0.861, n = 18 (negative geotaxis)*.*

We previously reported that resistance to Ma549 among 188 DGRP lines was negatively correlated with sleep duration at night in males (r = − 0.32, *p* < 0.0001, n = 156) and females (r = − 0.28, *p* = 0.0004, n = 156)^5^. Conversely, there was a positive association between resistance and the number of sleep bouts in males (r = 0.25, *p* = 0.0018, n = 156) and females (r = 0.24, *p* = 0.0028, n = 156)^5^. Compared to the total population, the resistance of the 20 divergent subset to Ma549 was even more closely associated with the number of nocturnal sleep bouts (males r = 0.67, *p* = 0.0026, n = 18; females r = 0.67, *p* = 0.0021, n = 18) and negatively correlated with night sleep duration (males r = − 0.71., *p* = 0.0009, n = 18; females r = − 0.74, *p* = 0.0005, n = 18). Hence, compared to the general population, there is a stronger trend for the 10 most resistant DGRP flies to have more sleep bouts than the 10 most susceptible DGRP flies, but these bouts are shorter and total sleep time is less. This trend was retained for *E. muscae,* but to a lesser degree, and falling short of significance, with the number of nocturnal sleep bouts (males r = 0.29., *p* = 0.2352, n = 18; females r = 0.35, *p* = 0.1483, n = 18) and negatively correlated with night sleep duration (males r = − 0.36, *p* = 0.1402, n = 18; females r = − 0.32, *p* = 0.1919, n = 18).

Looking at the data on a line-by-line basis shows why the associations are so weak. There are lines with increased levels of resistance to *E. muscae* and negative geotaxis, oxidative stress or sleep duration, but there are also resistant lines with moderate or low rankings for these indices, suggesting that there are no straightforward associations or trade-offs. Taking starvation stress as an example, as *E. muscae* colonizes the host’s body it will compete with it for resources^19^, so it makes intuitive sense that genotypes better able to tolerate starvation would have better tolerance to disease. Resistance to starvation is positively correlated with resistance to *E. muscae* in both males (r = 0.21, n = 20) and females (r = 0.34, n = 20). Although these values fall short of significance (*p* > 0.05) (Supplementary Fig S1p), they are higher than the correlations between resistance to Ma549 and starvation in males of (r = 0.17, n = 20) and females (r = − 0.03, n = 20). Resistance to starvation in the total population was only weakly correlated with the resistance of female flies to Ma549 (r = 0.16, *p* = 0.0335)^5^, indicating that *E. muscae* may cause greater nutrient stress to *Drosophila* than Ma549. However, on a line-by-line basis, DGRP lines RAL 38, RAL 48, RAL 443 and RAL 362 (highly resistant to both Ma549 and *E. muscae*), ranked 159, 28, 62 and 8 out of 203 DGRP lines for resistance to starvation. RAL 808 (Ma549 resistant, *E. muscae* susceptible), RAL 439 (Ma549 susceptible, *E. muscae* resistant) and RAL 227 (susceptible to both Ma549 and *E. muscae*) ranked 152, 162 and 149, respectively.

## Conclusion

Fungal-host interactions include both general broad host range and narrow host range pathogens. *E. muscae* is a dipteran specialist that naturally causes epizooitic outbreaks in *D. melanogaster*. Similar to broad host range ascomycete fungi, we identified considerable host genetic variation in resistance to *E. muscae* infection. However, we found that this variation is unlike ascomycetes, which kills different host genotypes with varying rapidity, but instead reflected considerable differences in the number of flies that died in a narrow window of time four to five days post-exposure. This reflects the unique *E. muscae* behavioral trait of killing flies in the hours leading to subjective sunset to ultimately maximize fungal dispersal. Despite differences in the co-evolutionary dynamics between *D. melanogaster* and *E. muscae*, versus other fungal pathogens such as *M. anisopliae*, flies showed cross-resistance to both fungi, indicative of generic anti-fungal defences. However, cross-resistance was greater between *M. anisopliae* and an opportunistic bacterial pathogen, *P. aeriginosa*, than between *M. anisopliae* and *E. muscae*. Also, *D. melanogaster* lines killed quickly by *M. anisopliae* Ma549 show greater variation in susceptibility to *E. muscae*, indicating that Ma549-susceptible individuals vary in evolution or retention of narrow anti-*E. muscae* mechanisms. Also, with the notable exception of starvation resistance, resistance to Ma549 and Pa14 correlated with non-specific physiological features such as sleep indices, to a greater extent than *E. muscae* infected flies, consistent with specific defenses being more important. This study demonstrates the continued utility of *Drosophila* for understanding host-fungus interactions, the clear potential for *Drosophila* to become a powerful in vivo comparative system to study the diversity of antifungal responses, and supports the utility of *E. muscae* as a model for studying varied aspects of host–pathogen interactions in the fly.

## Supplementary information

Supplementary Table 1.

Supplementary Table 2.

Supplementary figures.

## Data Availability

All data generated or analysed during this study are included in this published article (and its Supplementary Information files).
