# Supplementary material for: Genetic variation for resistance to the specific fly pathogen Entomophthora muscae
Source: Sci Rep. 2020 Aug 31;10:14284. doi: 10.1038/s41598-020-71262-w (PMC7459287; doi:10.1038/s41598-020-71262-w)
Supplement: Supplementary file 3 — Supplementary figures. [file 41598_2020_71262_MOESM3_ESM.pdf]

Title: Genetic variation for resistance to the specific fly pathogen *Entomophthora muscae*.

Jonathan B. Wang [1], Carolyn Elya[2], and Raymond J. St. Leger[1*]

[1]Department of Entomology, University of Maryland, College Park, Maryland 20742, USA; [2]Department of Organismic and Evolutionary Biology, Harvard University, Cambridge, Massachusetts 02138, USA.

Supplementary Figures:

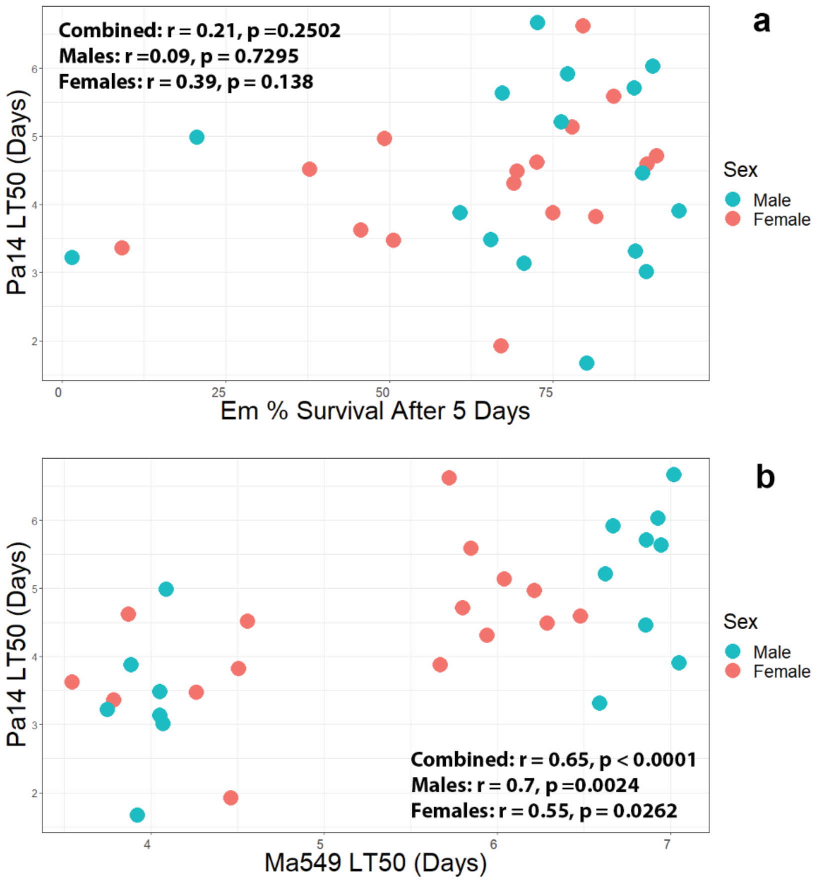

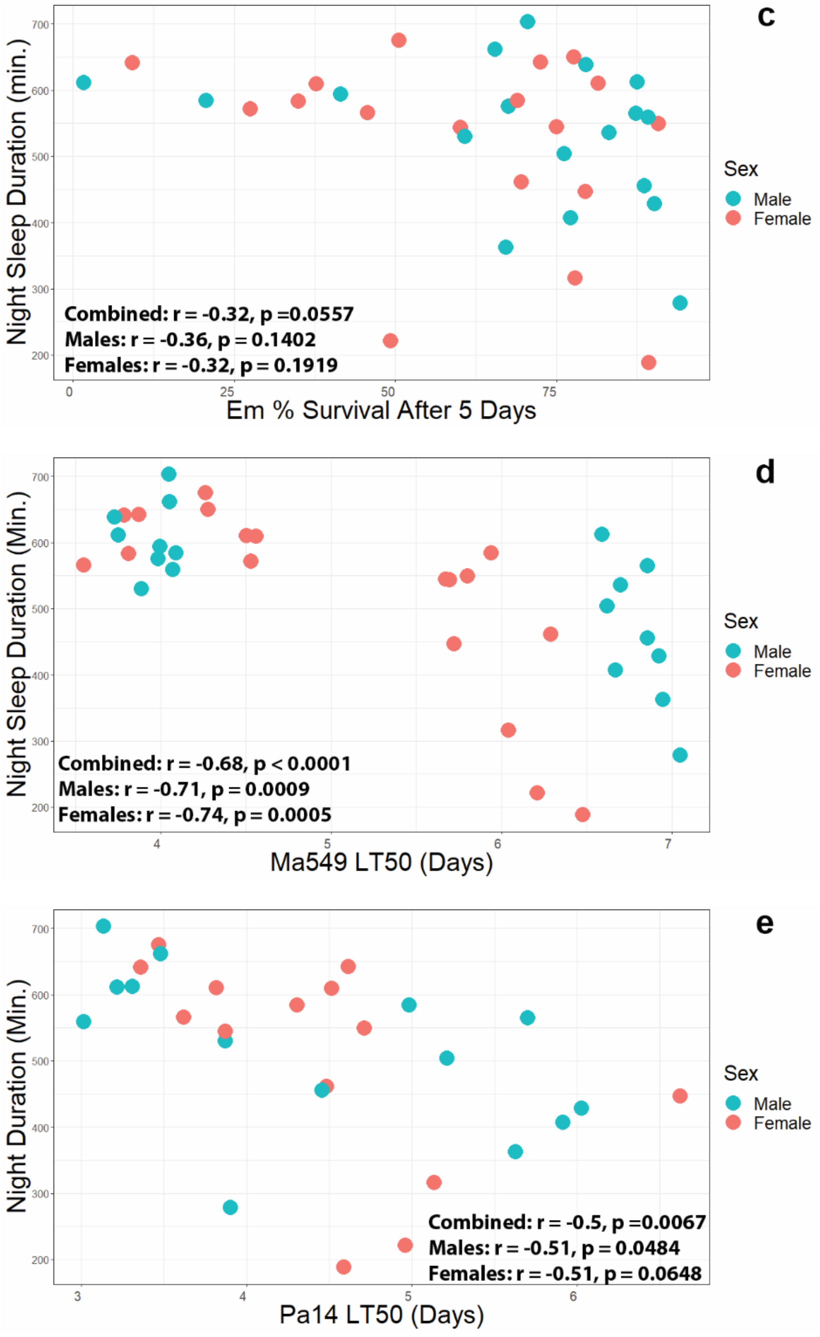
c
Night Sleep Duration (min.)
700
600
500
400
300
200
0
25
50
75
Em % Survival After 5 Days
Combined: r = -0.32, p =0.0557
Males: r = -0.36, p = 0.1402
Females: r = -0.32, p = 0.1919
Sex
Male
Female
d
Night Sleep Duration (Min.)
700
600
500
400
300
200
4
5
6
7
Ma549 LT50 (Days)
Combined: r = -0.68, p < 0.0001
Males: r = -0.71, p = 0.0009
Females: r = -0.74, p = 0.0005
Sex
Male
Female
e
Night Duration (Min.)
700
600
500
400
300
200
3
4
5
6
Pa14 LT50 (Days)
Combined: r = -0.5, p =0.0067
Males: r = -0.51, p = 0.0484
Females: r = -0.51, p = 0.0648
Sex
Male
Female

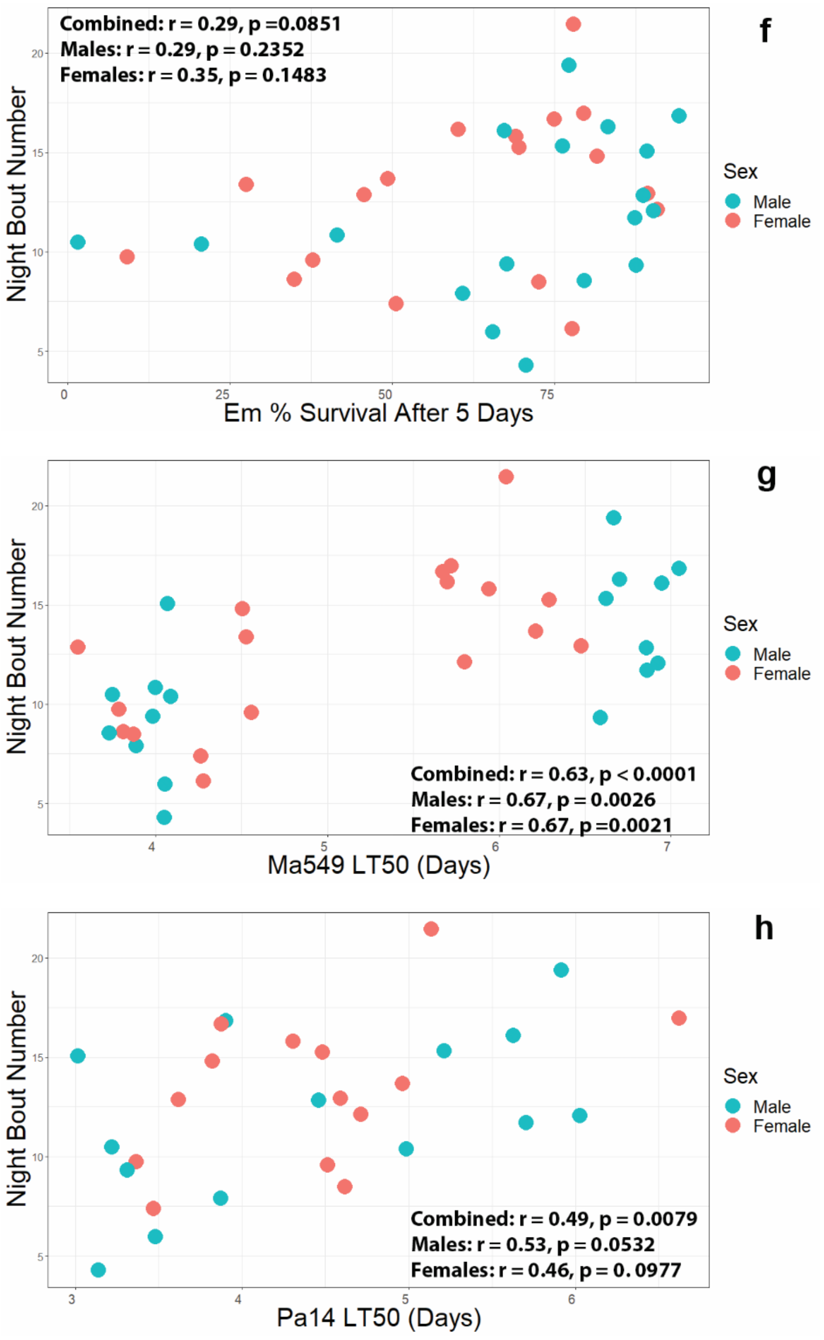
f
Combined: r = 0.29, p =0.0851
Males: r = 0.29, p = 0.2352
Females: r = 0.35, p = 0.1483
Night Bout Number
Em % Survival After 5 Days
Sex
Male
Female
g
Combined: r = 0.63, p < 0.0001
Males: r = 0.67, p = 0.0026
Females: r = 0.67, p =0.0021
Night Bout Number
Ma549 LT50 (Days)
Sex
Male
Female
h
Combined: r = 0.49, p = 0.0079
Males: r = 0.53, p = 0.0532
Females: r = 0.46, p = 0. 0977
Night Bout Number
Pa14 LT50 (Days)
Sex
Male
Female

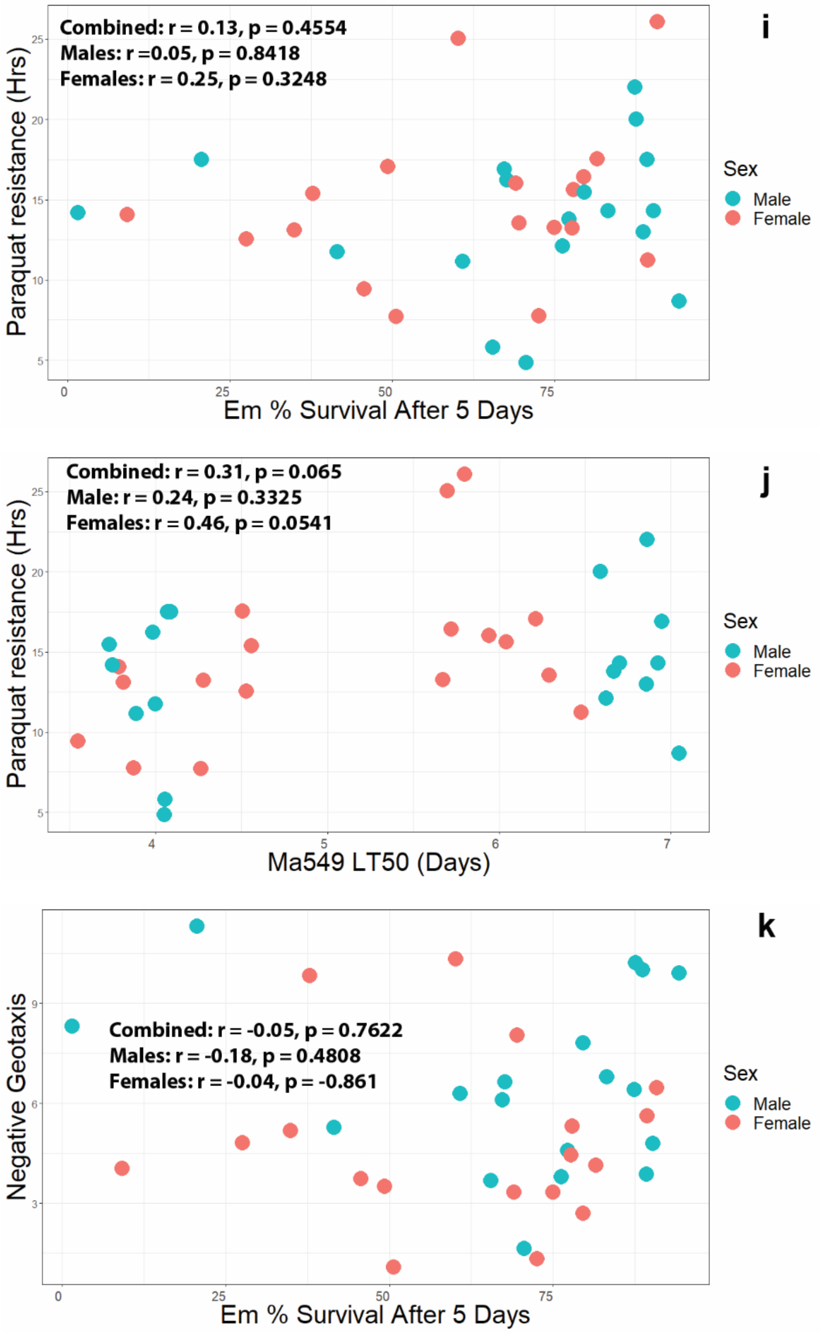
i
Combined: r = 0.13, p = 0.4554
Males: r =0.05, p = 0.8418
Females: r = 0.25, p = 0.3248
Paraquat resistance (Hrs)
Em % Survival After 5 Days
Sex
Male
Female
j
Combined: r = 0.31, p = 0.065
Male: r = 0.24, p = 0.3325
Females: r = 0.46, p = 0.0541
Paraquat resistance (Hrs)
Ma549 LT50 (Days)
Sex
Male
Female
k
Combined: r = -0.05, p = 0.7622
Males: r = -0.18, p = 0.4808
Females: r = -0.04, p = -0.861
Negative Geotaxis
Em % Survival After 5 Days
Sex
Male
Female

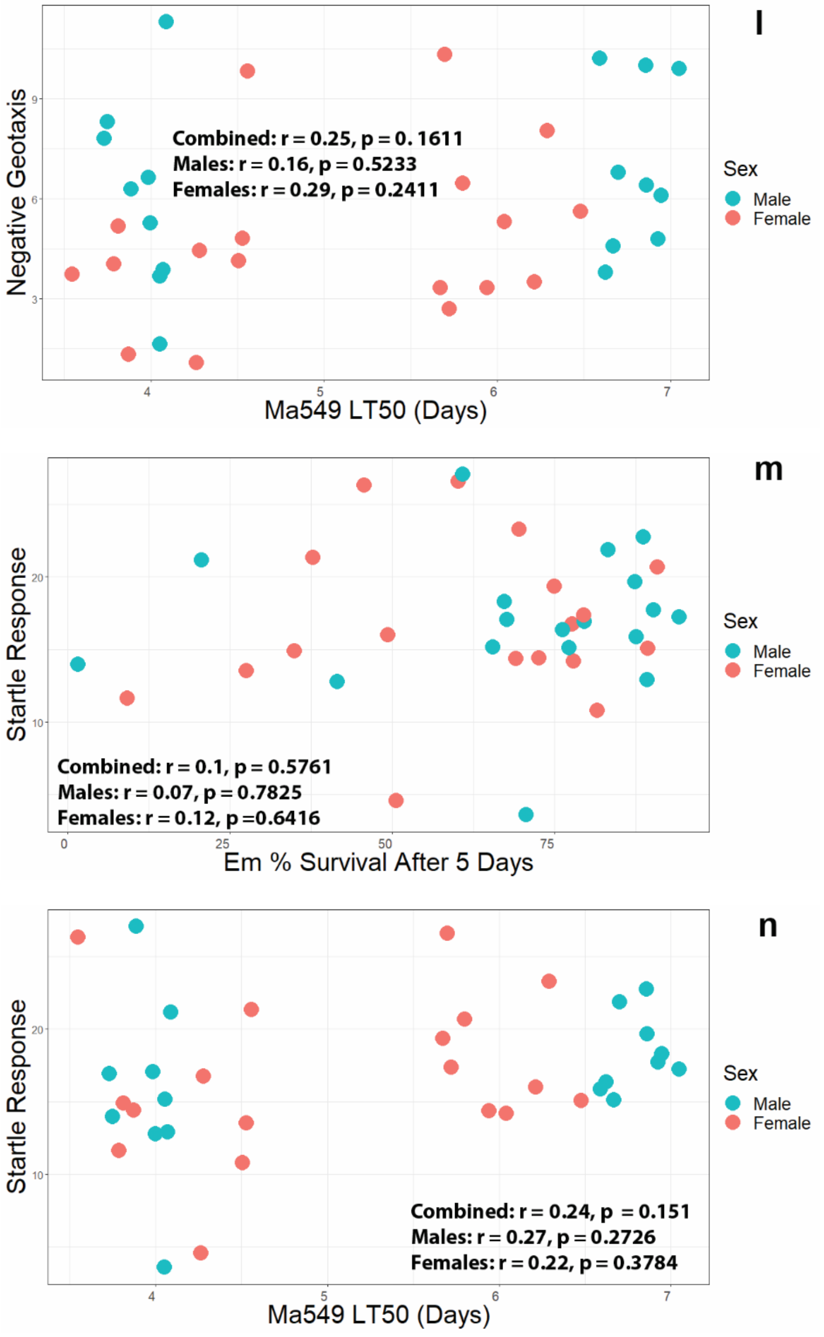
l
Negative Geotaxis
Combined: r = 0.25, p = 0. 1611
Males: r = 0.16, p = 0.5233
Females: r = 0.29, p = 0.2411
Ma549 LT50 (Days)
Sex
Male
Female
m
Startle Response
Combined: r = 0.1, p = 0.5761
Males: r = 0.07, p = 0.7825
Females: r = 0.12, p =0.6416
Em % Survival After 5 Days
Sex
Male
Female
n
Startle Response
Combined: r = 0.24, p = 0.151
Males: r = 0.27, p = 0.2726
Females: r = 0.22, p = 0.3784
Ma549 LT50 (Days)
Sex
Male
Female

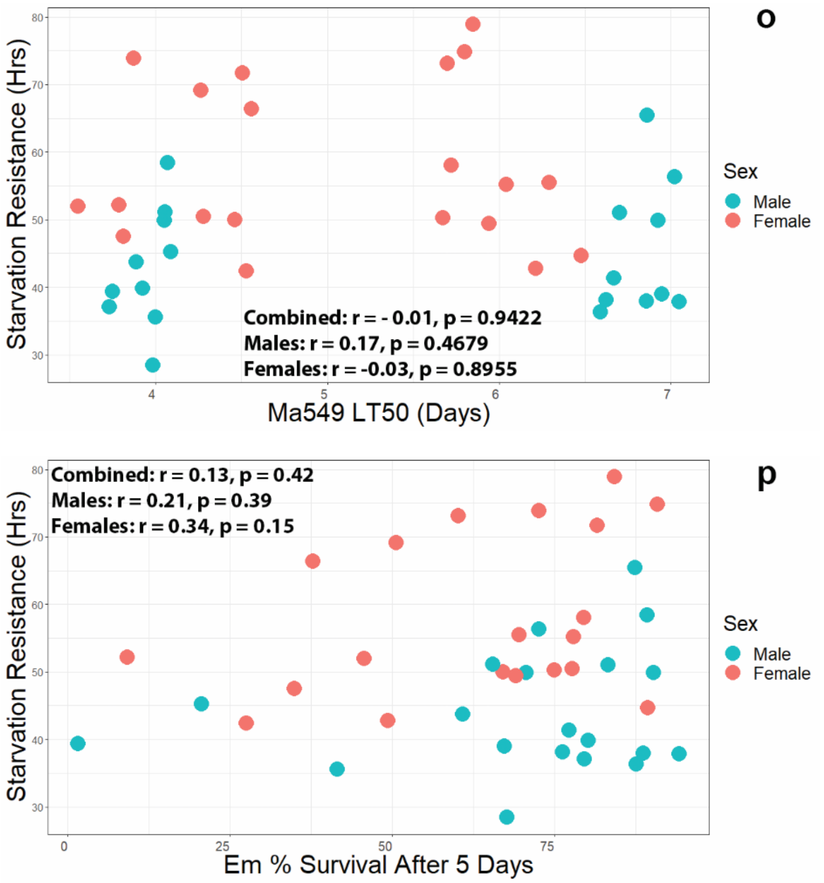

Supplementary Figure S1: Correlation graphs of phenotypes. (a) Positive correlation of % survival (5 days) among the DGRP lines between male and female flies infected with *E. muscae* and diverse phenotypes (b-p). *Pseudomonas aeruginosa* (Pa14) and Ma549 LT_{50} values were obtained from Wang et al., 2017[1], sleep data from Harbison et al., 2014[2], paraquat resistance from Weber et al., 2012[3], negative geotaxis and startle response data from Jordan et al., 2012[4], and starvation data from Mackay et al., 2012[5].

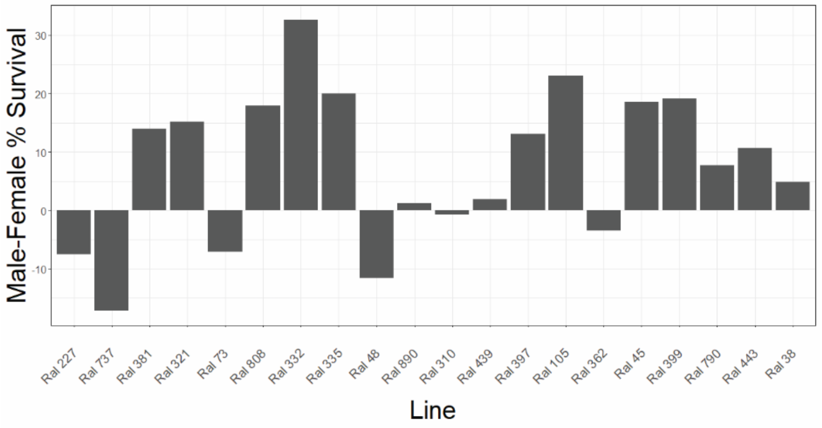

Supplementary Figure S2: Percent survival differences between male and female flies in DGRP lines infected by *E. muscae.* Flies were ordered (based on male percent survival) from the most susceptible (left) to the most resistant (right). Female percent survival was subtracted from male percent survival so positive values indicate lines where males are more resistant.

Supplementary References

1. Wang, J. B., Lu, H.-L. & St. Leger, R. J. The genetic basis for variation in resistance to infection in the *Drosophila melanogaster* genetic reference panel. *PLoS Pathog.* **13**, (2017). e1006260
2. Harbison, S. T., McCoy, L. J. & Mackay, T. F. C. Genome-wide association study of sleep in *Drosophila melanogaster*. *BMC Genomics* **14**, 281 (2013). doi:10.1186/1471-2164-14-281
3. Weber, A. L. *et al.* Genome-Wide Association Analysis of Oxidative Stress Resistance in *Drosophila melanogaster*. *PLoS One* **7**, e34745 (2012).
4. Jordan, K. W. *et al.* Genome-wide association for sensitivity to chronic oxidative stress in Drosophila melanogaster. *PLoS One* (2012). doi:10.1371/journal.pone.0038722
5. Mackay, T. F. C. *et al.* The *Drosophila melanogaster* Genetic Reference Panel. *Nature* **482**, 173–178 (2012).
6. Durham, M. F., Magwire, M. M., Stone, E. A. & Leips, J. Genome-wide analysis in *Drosophila* reveals age-specific effects of SNPs on fitness traits. *Nat. Commun.* **5**, 1-8 (2014).
